# A Case of Brown-Vialetto-Van Laere Syndrome Due To a Novel Mutation in *SLC52A3* Gene: Clinical Course and Response to Riboflavin

**DOI:** 10.1177/2329048X17725610

**Published:** 2017-08-22

**Authors:** Venkatraman Thulasi, Aravindhan Veerapandiyan, Beth A. Pletcher, Chun M. Tong, Xue Ming

**Affiliations:** 1 Division of Pediatric Neurology, Department of Neurology, Rutgers New Jersey Medical School, Newark, NJ, USA; 2 Division of Clinical Genetics, Department of Pediatrics, Rutgers New Jersey Medical School, Newark, NJ, USA

**Keywords:** riboflavin transporter deficiency, Brown-Vialetto-Van Laere syndrome, *SLC52A3*, pontobulbar palsy, ataxia

## Abstract

Brown-Vialetto-Van Laere syndrome is a rare disorder characterized by motor, sensory, and cranial neuronopathies, associated with mutations in *SLC52A2* and *SLC52A3* genes that code for human riboflavin transporters RFVT2 and RFVT3, respectively. The authors describe the clinical course of a 6-year-old girl with Brown-Vialetto-Van Laere syndrome and a novel homozygous mutation c.1156T>C in the *SLC52A3* gene, who presented at the age of 2.5 years with progressive brain stem dysfunction including ptosis, facial weakness, hearing loss, dysphagia, anarthria with bilateral vocal cord paralysis, and ataxic gait. She subsequently developed respiratory failure requiring tracheostomy and worsening dysphagia necessitating a gastrostomy. Following riboflavin supplementation, resolution of facial diplegia and ataxia, improvements in ptosis, and bulbar function including vocalization and respiration were noted. However, her sensorineural hearing loss remained unchanged. Similar to other cases of Brown-Vialetto-Van Laere syndrome, our patient responded favorably to early riboflavin supplementation with significant but not complete neurologic recovery.

Brown-Vialetto-Van Laere syndrome is a rare autosomal recessive neurological disorder characterized by axial and appendicular weakness, sensory neuronopathy, gait ataxia, respiratory difficulties, bulbar palsy, sensorineural hearing loss, optic atrophy, and facial weakness. Age of onset varies from infancy to adulthood and, if untreated, can be fatal.^1,2^ Mutations in the riboflavin transporter genes *SLC52A2* and *SLC52A3* coding for human riboflavin transporters RFVT2 and RFVT3, respectively, have been associated with Brown-Vialetto-Van Laere syndrome.^1–3^ To date, 34 patients with molecular diagnosis of Brown-Vialetto-Van Laere syndrome caused by mutations in the *SLC52A3* gene have been reported.^2,4^ High-dose riboflavin supplementation has shown to significantly improve clinical outcomes.^2,4,5^ In this report, the authors describe the clinical course and responsiveness to riboflavin of a patient with Brown-Vialetto-Van Laere syndrome who was found to have a novel missense mutation in *SLC52A3.*

## Case Report

Our patient is a 6-year-old Hispanic girl who presented at the age of 2.5 years with speech regression, ptosis, gait abnormality, and choking. She was born to nonconsanguineous Ecuadorian parents after an uneventful pregnancy. Newborn hearing screen was unremarkable. Developmental domains were attained appropriately until 6 months prior to the presentation. She had her first words at the age of 10 months, 12- to 15-word vocabulary at the age of 1.5 years, and started putting 2 words together at 2 years of age. Speech regression was insidious, but at the time of presentation, she was completely nonverbal yet retained her receptive language skills. She developed progressive bilateral ptosis, and gait ataxia over 2 months, and experienced several episodes of choking in the week prior to the presentation. A brother who had similar symptoms deteriorated over a 3-month period of time and died at the age of 4.5 years from respiratory failure. Neurological examination demonstrated anarthria, bilateral near complete ptosis (left worse than right), facial diplegia, weak gag reflex, mild generalized weakness, mild spasticity of lower extremities with no contractures, symmetric brisk deep tendon reflexes with ankle clonus, truncal ataxia, and wide-based ataxic gait. Sensory examination showed responsiveness to light touch and pain. Position and vibration sense could not be reliably ascertained due to patient’s age and inability to talk. She was noted to have increased inspiratory effort. Testing revealed bilateral sensorineural hearing loss (brain stem auditory evoked response and behavioral audiometry) and moderate-to-severe oropharyngeal dysphagia, with a normal ophthalmologic examination. There was no evidence of nonverbal cognitive deficits.

Serum amino acids, acylcarnitine profile including short-, medium-, and long-chain acylcarnitines, lactic acid, ammonia, and cerebrospinal fluid analysis were within normal limits. Blood riboflavin level was normal at 171 μg/L (137-370). Magnetic resonance imaging of the brain without contrast, visual-evoked response, and electroencephalogram were normal. Sequencing of the *SLC52A3* gene revealed a homozygous missense mutation p.Cys386Arg (c.1156T>C) in exon 4. She was started on oral riboflavin supplementation at a dose of 25 mg/kg/d. Improvement in ptosis, facial diplegia, gait, and activity level was noted within 2 to 3 days. The parents refused gastrostomy and respiratory assistance. She was discharged on riboflavin and a modified diet.

Two weeks later, the patient was hospitalized after an episode of choking while being fed pureed food. She underwent percutaneous endoscopic gastrostomy tube placement. Her respiratory insufficiency worsened requiring bilevel positive airway pressure during sleep. Flexible laryngoscopic examination revealed bilateral vocal cord dysfunction. She remained neurologically stable for the following 3 months until she was hospitalized for respiratory failure necessitating a tracheostomy. She was continued on riboflavin supplementation, percutaneous endoscopic gastrostomy feeding, physical, occupational, and speech therapies. Facial weakness, ataxic gait, and motor functions continued to steadily improve. At the age of 4 years, she had mild residual left ptosis and ataxia, was able to tolerate small amounts of soft food by mouth, and had no facial or motor weakness, however, her vocal cord paralysis and hearing did not improve. Polysomnography showed obstructive and central sleep apneas, with an apnea hypopnea index of 11.2 per hour. Mechanical ventilatory assistance during sleep was initiated. Riboflavin dose was increased to 30 mg/kg/d.

Our patient’s neurological status plateaued for the next year. Riboflavin was increased to 40 mg/kg/d at the age of 5 years and further improvements were noted in ptosis, gait, and vocalization. Riboflavin dose was increased to a current dose of 60 mg/kg/d with no adverse effects. Most recent neurological examination at the age of 6 years showed normal gait, normal muscle tone and deep tendon reflexes, no tremor, and mild left ptosis. She was able to laugh and scream while playing but was unable to speak clear words. Her respiratory effort had improved, but sensorineural hearing loss remained unchanged. She attends a school for hearing-impaired children.

## Discussion

**Figure 1.**
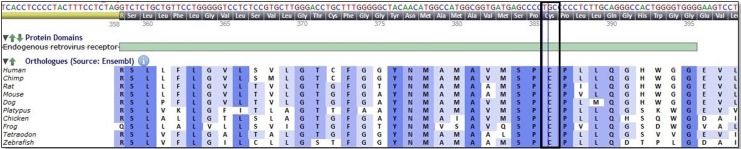
Picture illustrating the conservation of cysteine at position 386 (p.Cys386 residue) in *SLC52A3* gene among species.

Brown-Vialetto-Van Laere syndrome is a rare neurological disease with progressive pontobulbar palsy, sensorineural hearing loss, and respiratory compromise, associated with compound heterozygous or homozygous mutations in the *SLC52A3* and *SLC52A2* genes.^2,6^ A recent review article reported 70 patients with a molecular diagnosis of riboflavin transporter deficiency, and 33 of them had mutations in the *SLC52A3* gene.^2^ To the best of our knowledge, the c.1156T>C mutation in exon 4 of *SLC52A3* found in our patient has not been reported in the literature. This missense mutation causes replacement of the amino acid cysteine by arginine at codon 386. The amino acid substitution is predicted to be “probably damaging” by the polymorphism phenotyping tool (Polyphen-2). The p.Cys386 residue in *SLC52A3* gene has been highly conserved among species during evolution (Alamut Visual 2.9, http://www.interactive-biosoftware.com/alamut-visual; Figure 1). Moreover, substitution of a nearby amino acid (p.Gly375Asp) has been reported to be causative for Brown-Vialetto-Van Laere syndrome.^7^ The authors speculate that the homozygous mutation affecting this highly conserved region is associated with the Brown-Vialetto-Van Laere syndrome phenotype in our patient.

Mutations in *SLC52A3* gene affect the expression of the riboflavin transporter RFVT3, which is mainly expressed in the small intestine and moderately expressed in the central nervous system. RFVT3 is thought to play a major role in the intestinal absorption of riboflavin.^8,9^ Although, low riboflavin levels and abnormalities in acylcarnitine profiles were initially identified in patients with a molecular diagnosis of Brown-Vialetto-Van Laere syndrome affecting RFVT3,^10^ further studies have clarified that such abnormalities are not seen in all patients.^2^ Interestingly, even patients with normal baseline riboflavin levels have shown improvement with riboflavin therapy.^1^ Our patient had normal riboflavin level and showed substantial clinical improvement with riboflavin supplementation.

Riboflavin supplementation has been shown to decrease mortality in patients with Brown-Vialetto-Van Laere syndrome.^1,2^ A recent review by Jaeger and Bosch demonstrated that all patients who were not treated with riboflavin showed gradual decline over time.^2^ This review also showed that the majority of the patients (8/13 patients) with defects in RFVT3 who were treated with riboflavin improved, 2 patients remained stable, and 3 showed no response. Dosage used in these patients range from 7 to 60 mg/kg/d. Although some patients show improvement within days of riboflavin supplementation, others with more severe symptoms have a more gradual recovery over months.^1,2^ Our patient initially exhibited a rapid improvement that began within 24 hours of initiating riboflavin treatment, followed by a plateauing of abilities over a 1-year period, and then continued improvement with dose increments. There was resolution of ptosis, facial diplegia, weakness, and ataxia and improvement in bulbar function, including vocalization, and respiration over a 3.5-year span. The mean age of death for patients presenting under the age of 4 years and left untreated is 1.9 years, and the primary cause of death is respiratory insufficiency.^1^ Fortunately, due to early diagnosis and supplementation with riboflavin, our patient has survived to her current age of 6 years. Thus, treating physicians are urged to initiate riboflavin therapy as soon as there is suspicion for Brown-Vialetto-Van Laere syndrome while awaiting genetic confirmation and to continue supplementation even in the absence of initial treatment response.

This case identifies a novel mutation for Brown-Vialetto-Van Laere syndrome and emphasizes the need for early diagnosis and management of symptoms which can lead to improved clinical outcomes in patients with this disease. It is important for physicians to consider this rare neurologic disease when evaluating patients with similar symptoms.
